# Mouse SNP Miner: an annotated database of mouse functional single nucleotide polymorphisms

**DOI:** 10.1186/1471-2164-8-24

**Published:** 2007-01-21

**Authors:** Eli Reuveni, Vasily E Ramensky, Cornelius Gross

**Affiliations:** 1 Mouse Biology Unit, EMBL, Via Ramarini 32, 00016 Monterotondo, Italy; 2 Engelhardt Institute of Molecular Biology, Vavilova 32, 119991 Moscow, Russia

## Abstract

**Background:**

The mapping of quantitative trait loci in rat and mouse has been extremely successful in identifying chromosomal regions associated with human disease-related phenotypes. However, identifying the specific phenotype-causing DNA sequence variations within a quantitative trait locus has been much more difficult. The recent availability of genomic sequence from several mouse inbred strains (including C57BL/6J, 129X1/SvJ, 129S1/SvImJ, A/J, and DBA/2J) has made it possible to catalog DNA sequence differences within a quantitative trait locus derived from crosses between these strains. However, even for well-defined quantitative trait loci (<10 Mb) the identification of candidate functional DNA sequence changes remains challenging due to the high density of sequence variation between strains.

**Description:**

To help identify functional DNA sequence variations within quantitative trait loci we have used the Ensembl annotated genome sequence to compile a database of mouse single nucleotide polymorphisms (SNPs) that are predicted to cause missense, nonsense, frameshift, or splice site mutations (available at ). For missense mutations we have used the PolyPhen and PANTHER algorithms to predict whether amino acid changes are likely to disrupt protein function.

**Conclusion:**

We have developed a database of mouse SNPs predicted to cause missense, nonsense, frameshift, and splice-site mutations. Our analysis revealed that 20% and 14% of missense SNPs are likely to be deleterious according to PolyPhen and PANTHER, respectively, and 6% are considered deleterious by both algorithms. The database also provides gene expression and functional annotations from the Symatlas, Gene Ontology, and OMIM databases to further assess candidate phenotype-causing mutations. To demonstrate its utility, we show that Mouse SNP Miner successfully finds a previously identified candidate SNP in the taste receptor, Tas1r3, that underlies sucrose preference in the C57BL/6J strain. We also use Mouse SNP Miner to derive a list of candidate phenotype-causing mutations within a previously uncharacterized QTL for response to morphine in the 129/Sv strain.

## Background

The laboratory mouse is a powerful model for studying the genetic determinants of human disease-related phenotypes. One way to study genetic modifiers of such phenotypes is to take advantage of genetic variations existing between mouse inbred strains to map chromosomal regions, or loci, that are associated with quantitative traits, so-called quantitative trait loci (QTL). Over 2,000 QTL are listed in the Mouse Genome Informatics database [1], and many of these are relevant to human disease.

However, the identification of causal functional DNA polymorphisms underlying QTL has remained problematic, with such variations having been convincingly identified for no more than 9 QTL [2]. This difficulty stems largely from the considerable size of typical QTL (10–50 Mb) and the large number of sequence variations that lie within such a region. In most cases, gene discovery for QTL is carried out by congenic mapping to narrow down the QTL region to less than 5 Mb, followed by gene expression and coding sequence analysis of each gene in the interval to narrow down a list of candidate genes. Finally, the most promising candidates are tested by genetic complementation or epistasis in transgenic animals. Several methods are helping to speed the process of narrowing down QTL to <5 Mb, including mapping in chromosome substitution strains [3], heterogeneous stocks [4], and advanced intercrosses [5]. The construction of large panels of recombinant inbred strains (>500 lines) are in progress and promise to facilitate rapid high-resolution mapping [6].

Recently, the nearly complete genome sequences of four mouse inbred strains (129X1/SvJ, 129S1/SvImJ, A/J, DBA/2J; [7]) joined the public reference sequence (C57BL/6J; [8]) in open-access genome databases. Complete knowledge of the DNA sequence variation between strains allows for a systematic search of all candidate sequence polymorphisms within a QTL for putative functional polymorphisms [9-11]. When coupled with gene annotation, this information can be used to identify polymorphisms likely to cause changes in gene function and thus likely to contribute to the QTL. Single nucleotide polymorphisms (SNPs) that cause nonsense or missense mutations can be reliably identified and often are responsible for severe changes in protein function. Among 9 identified causal QTL mutations, 2 are missense mutations, 2 are non-sense mutations, and 1 is a frameshift mutation demonstrating that these classes of overt coding sequence mutations contribute to QTL phenotypes, at least for QTL of large effect size [2]. It is unclear in this point what fraction of the >2000 reported mouse QTL are caused by overt coding sequence mutations, and it is likely that functional non-coding mutations contribute significantly to these traits. However, the success of finding at least some functional coding sequence mutations underlying QTL coupled with the much greater reliability with which such mutations can be identified at the present time suggests that searching for functional coding mutations is a worthwhile initial approach for QTL analysis.

Several authors have used the Celera Discovery System [12] database to identify putative missense and nonsense SNPs within QTL [13-16]. Beginning with Mouse Build 126, December 2006, the public SNP repository, dbSNP [17], has incorporated all Celera sequence reads from 129X1/SvJ, 129S1/SvImJ, A/J, and DBA/2J and thus provides free access to genomic sequence from several inbred strains. Both the Ensembl and NCBI genome portals offer search tools that allow comparison of SNPs between mouse inbred strains, called TranscriptSNPView [18] and SNPviewer [19], respectively. SNP calls from dbSNP are mapped onto the Ensembl or NCBI annotated mouse genome to make predictions about the functional consequence of each SNP. We chose to use genome annotations from Ensembl because it provides open access to its MySQL database and associated API interface [20]. Although these databases allow searching of SNPs according to specific criteria (e.g., chromosome location, pair-wise strain comparison, etc.), neither of them provides additional annotations for SNPs, such as deleterious/non-deleterious for missense mutations, gene expression data, or relevance to human disease.

To address this need, we sought to build a database of mouse inbred strain SNPs predicted to cause functional changes in protein sequence, including missense, nonsense, and splice site mutations, that could be easily searched by strain, type, chromosomal location and functional consequence. In addition, we applied the bioinformatics algorithms PolyPhen [21] and PANTHER [22] to predict whether missense mutations among these SNPs were likely to disrupt protein function. PolyPhen analysis of human SNPs showed that 17% of a selected set of 9,165 human non-synonymous coding SNPs are 'possibly damaging' and 13% are 'probably damaging' to protein function. These data confirmed earlier reports suggesting that only a small fraction of missense mutations interfere with protein function. We reasoned that a similar analysis of mouse missense SNPs could help to narrow down candidate causal SNPs within QTL. The PANTHER algorithm offers a complementary functional assessment of putative missense SNPs [22] and a comparison of PolyPhen and PANTHER predictions for human missense SNPs demonstrated a close correlation between these predictions and empirical assessments of protein activity [23]. The recent deposition of Celera sequence reads from the 129X1/SvJ, 129S1/SvImJ, A/J, and DBA/2J mouse inbred strains into the public domain has made it possible for us to develop a database of mouse SNPs that incorporates both Polyphen and PANTHER missense SNP functional predictions as well as other gene function annotations.

## Construction and content

**Figure 1:**
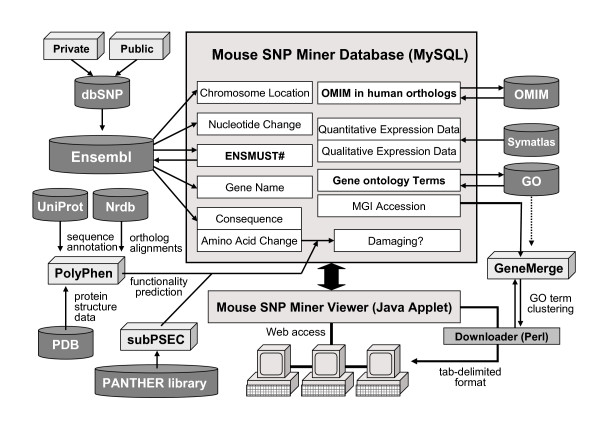
**Mouse SNP Miner database structure**. The core database consists of a MySQL relational database containing information associated with predicted functional mouse SNPs from a selected set of mouse inbred strains. A web-based Java Applet module allows querying, visualization, and downloading of information from the database. Basic information about SNP sequence, location, functional consequence, and associated transcript are derived from private and public sequencing efforts via the dbSNP mouse polymorphism collection mapped onto the annotated Ensembl genome. Additional SNP information was extracted from the OMIM, Symatlas, and GO databases. GO clustering by GeneMerge is queried directly by the Applet viewer prior to downloading. PolyPhen assessment of missense mutation consequence was based on Nrdb orthologous protein alignments, PDB structure information, and protein functional annotation from Uniprot. PANTHER assessment of missense mutation consequence was based on a set of HMM protein alignments. Bold font and arrows pointing out of the database indicate the existence of direct web links from our database to associated database entries.

To assemble mouse SNPs for our database, we used Perl scripts and MySQL queries to retrieve SNPs from the Ensembl 'core', 'variation' and 'mart' databases (Ensembl v37, February 2006 and dbSNP v125) that differ between 28 commonly used inbred strains. Ensembl annotations were used to classify SNPs as putative coding or non-coding mutations and for coding variants, as synonymous, non-synonymous (missense), STOP-gained, STOP-lost, splice-site, or frameshift. For non-synonymous mutations, information about the assignment of amino acid variants to the corresponding mouse strain was not available in Ensembl v37 and had to be derived by mapping the chromosomal location of the mutation onto a translation of the associated transcript. Related strains were grouped together so as to allow convenient searching for SNPs differing between strain families (i.e. C57% = 'C57BL/6J', 'C57BL/10J', 'C57BL/10SnJ', etc.). We have also incorporated sequence coverage details when available in order to confer SNP reliability. Table 1 summarizes the number of putative functional SNPs for three strain comparisons, C57BL/6J vs. DBA/2J, C57BL/6J vs. 129S1/SvImJ, and C57BL/6J vs. A/J. For each gene in the database, annotations from several databases were extracted and deposited in our database: 1) gene ontology (GO) categories [24], 2) Symatlas gene expression data [25], and 3) OMIM human disease phenotypes [26]. Symatlas data include both categorical, 'absent'/'present' calls, as well as quantitative Affymetrix values for a large set of embryonic, neonatal and adult mouse tissues. A schematic of the database is shown in Figure 1.

For missense mutations, functionality prediction was performed with the algorithms PolyPhen [21] v1.12 (based on March 2006 releases of UniProt [27], NCBI nrdb [28], PDB [29] and DSSP databases [30]) and PANTHER [22] (based on PANTHER HMM library v6.0). PolyPhen is a computational tool for identification of potentially functional nsSNPs. Predictions are based on a combination of phylogenetic, structural and sequence annotation information characterizing a substitution and its position in the protein. For a given amino acid variation, PolyPhen performs several steps: (a) extraction of sequence-based features of the substitution site from the UniProt database, (b) calculation of profile scores for two amino acid variants, (c) calculation of structural parameters and contacts of a substituted residue. PANTHER Version 6.0 library contains a set of over 5,000 protein families and about 30,000 subfamilies derived from those families, each represented by a multiple sequence alignment and Hidden Markov Model (HMM). The subfamilies are a subset of selected proteins that can be associated with functional classification (cellular process and molecular function) using manual expert curation. Missense SNPs can be scored against these HMM families to estimate their likelihood of disrupting conserved amino acid elements, and thus protein function [22].

PolyPhen scores were classified as 'benign', 'possibly damaging', or 'probably damaging' and PANTHER scores as 'non-deleterious' or 'deleterious' following previously described criteria [22]. For both algorithms, a label of 'unknown' was assigned in cases where insufficient annotations or orthologs existed to draw conclusions about functionality of amino acid variants. The PANTHER algorithm tended to result in a greater number of 'unknown' variants presumably because the HMM library search was more stringent that the BLAST search used by PolyPhen and thus more variants lacked sufficient homologs for comparative analysis. A summary of PolyPhen and PANTHER predictions for three inbred strain comparisons is shown in Table 2. Of a total of 27,143 missense SNPs in the database, PolyPhen predicted 5,302 (20%) to be 'possibly damaging' or 'probably damaging' (deleterious), 19,390 (72%) as 'benign' (non-deleterious), and 2,260 (8%) as 'unknown'. PANTHER predicted 3,770 (14%) to be 'deleterious', 13553 (50%) as 'non-deleterious', and 9444 (35%) as 'unknown'. More than 6% of missense SNPs were classified as deleterious, and 42% as non-deleterious by both PolyPhen and PANTHER (Table 3). The smaller fraction of PolyPhen 'deleterious' calls that are considered 'deleterious' by PANTHER is likely at least in part to be due to the greater number of 'unknown' PANTHER calls. Finally, a search of the OMIM database [26] for genes harboring predicted damaging SNPs (splice site, frameshift, STOP-gain, STOP-lost, and probably or possibly damaging missense as called by PolyPhen) retrieved entries for 803 genes (Table 4). This finding suggests that genetic variation among mouse inbred strains may serve as a promising tools to model human disease-relevant phenotypes.

**Figure 2:**
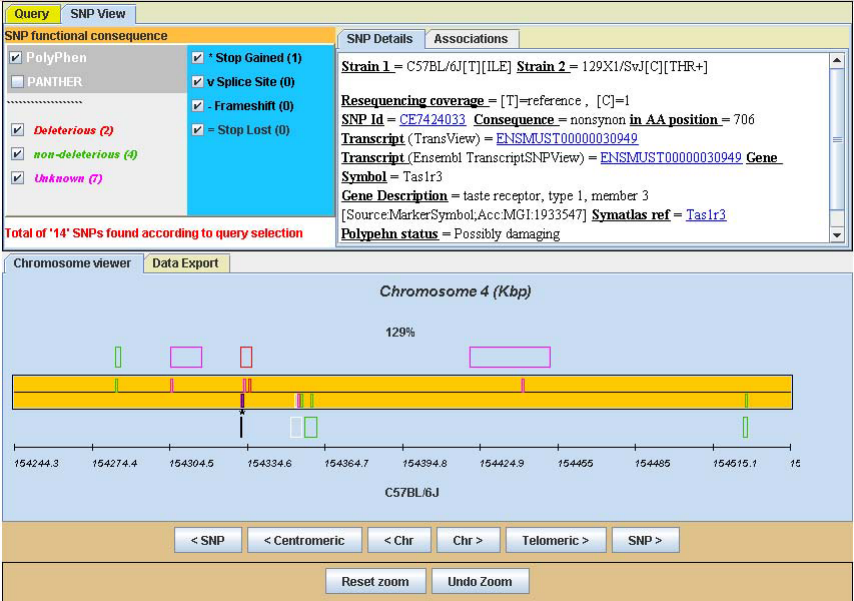
**Web-based access to Mouse SNP Miner database**. (**not shown**) 'SNP Query' mode allows selection of strain or strain group for comparison. Searching can be constrained by chromosomal interval, gene name, SNP accession number, and presence of human ortholog in OMIM database. QTL from the MGI database can be searched by name or keyword and associated chromosome intervals imported for convenient screening. (**shown**) 'SNP View' mode presents results from the search in a graphical format for convenient run-time scanning. SNPs in the interval are listed by functional consequence and PolyPhen/PANTHER prediction in the upper left and can be rapidly added or removed by clicking on the associated box. Boxes indicating transcripts and lines indicating SNPs are color and symbol coded by functional consequence in the graphical display. The placement of marks above and below the bar indicates transcripts in the forward and reverse strand, respectively. Clicking on a SNP causes detailed SNP information to be displayed in the 'Details' window above. The 'Associations' window displays GO, OMIM, and PolyPhen/PANTHER information and links for the selected SNP. Movement across the chromosome and between SNPs is facilitated by buttons at the bottom of the graphical display. In the example shown, a search has been performed for putative functional SNPs differing between C57BL/6J and all 129 strains (129% allows global searching of 129 strains) for the interval 153,482,802–154,678,264 bp on chromosome 4. A deleterious mutation (Ile706Thr) in the fourth transmembrane domain of Tas1r3 is highlighted.

All the above data were stored in a MySQL database available online via a web interface. A Java Applet allows searching of the SNP database by strain, functional type (e.g. missense, STOP-gain), functional consequence (e.g. deleterious, non-deleterious), chromosomal location, GO accession number, QTL symbol or name, and re-sequencing coverage (where available), and GO or OMIM keywords (Figure 2). Browsing of retrieved SNPs is facilitated by a graphical display in which retrieved SNPs are displayed chromosome-by-chromosome. Zooming is facilitated by directly selecting a region of the chromosome for enlargement. Buttons allow chromosome walking to the right or left, in-and-out zooming, and jumping between adjacent SNPs. Changes in SNP retrieval criteria are immediately reflected in the graphical display making it possible to quickly view different sets of SNPs for a given chromosomal region. Clicking on a SNP marker in the graphical display returns a summary of relevant SNP and gene annotations. Several links are provided within the SNP summary table, including links to the Ensembl SNP View, Ensembl Transcript View, Ensembl TranscriptSNPView, Symatlas expression data, GO terms and OMIM entries.

Annotations for a selected set of SNPs can be conveniently exported in tab-delimited format for offline analysis. The export summary file provides dbSNP accession numbers, functional type and consequence, gene accession description information, GO terms, and OMIM description (if available) for each SNP. In addition, Symatlas expression data for SNP-associated transcripts in the interval can be exported in tab-delimited format. Symatlas expression data is derived from Affymetrix microarray assessment of transcript abundance from over 40 mouse tissues [27]. Both quantitative expression data (adjusted signal intensity) as well as qualitative expression data (present/absent) are included to facilitate rapid assessments of transcript abundance in target tissues. Finally, a list of clustered gene ontology terms produced by the GeneMerge algorithm [31] can be exported in tab-delimited format. This file clusters all GO terms associated with a set of genes retrieved from the database and thus allows rapid identification of subsets of genes with overlapping GO terms.

## Utility

We assembled a database of predicted functional SNPs deriving from 28 mouse inbred strains. We used the bioinformatics algorithms PolyPhen and PANTHER to assess whether predicted missense mutations are likely to alter protein function. This database is intended to help in the identification of candidate functional SNPs underlying QTL between mouse inbred strains.

Two features make our database unique. First, we performed bioinformatics-based estimations of functional consequence for missense mutations using the PolyPhen and PANTHER algorithms. These estimations show that ~28% of missense coding SNPs are deleterious to protein function according to at least one of the two prediction algorithms and ~6% are deleterious according to both algorithms. These predictions can be used to help focus studies on those SNPs within a QTL that are most likely to alter protein function. Second, we have annotated functional SNPs with gene expression, GO, and OMIM data to allow searching and browsing by these criteria. The integration of these annotations into a single SNP repository facilitates the rapid scanning of SNPs within an interval of interest for candidate phenotype-causing mutations.

We assessed the utility of our database by using it to identify candidate phenotype-causing SNPs for one previously cloned and one as yet uncloned mouse QTL. Free choice sucrose preference varies significantly between mouse inbred strains and a QTL determining sucrose preference between high and low sucrose preferring strains (e.g. C57BL/6J and 129P3/J, respectively) was localized to the 1.2 Mb interval between markers D4Mit256 and 139J18 [32]. Using congenic 129.B6 mice in which a 194 kb genomic segment from the C57BL/6J strain was introgressed into the 129P3/J strain and which displayed high sucrose preference, a region containing twelve predicted genes was identified [32]. One of these genes, Tas1r3, encodes a taste receptor family member and contains several missense mutations (Thr55Ala, Ile60Thr) that segregate with sucrose preference among six inbred strains [32]. The genomic location, primary protein sequence, and existence of non-synonymous mutations were used as evidence that mutations in Tas1r3 underlie this QTL. Subsequently mice lacking Tas1r3 were engineered and confirmed to display decreased sucrose preference [33].

To test whether Mouse SNP Miner could be used to draw similar conclusions without the need for high resolution mapping using congenic mice, we analyzed putative functional SNPs between C57BL/6J and 129 strains for the entire 1.2 Mb QTL between D4Mit256 and 139J18. Search of the Mouse SNP Miner database identified 14 putative functional SNPs differing between C57BL/6J and 129% (129% retrieves all 129 substrain SNPs) and lying in the interval from 153,482,802 to 154,678,264 Mb on chromosome 4 (Ensembl v36). Of 51 transcripts in the interval, 7 contained putative functional SNPs (Figure 2). One gene within the interval, matrix-remodelling associated protein 8 (Mxra8), contained a deleterious missense mutation (Tyr364His) in the 129X1/J strain. Symatlas data demonstrated that Mxra8 was widely expressed in the mouse, with highest expression in adult lung. An intronless Mxra8 pseudogene encoded on the reverse strand contained a putative premature termination codon (Trp14*) in the 129X1/J strain but Symatlas data suggested that the transcript was not detectably expressed even in tissue from C57BL/6J. As expected, the Tas1r3 gene contained a mutation (Ile706Thr) in the 129X1/J strain that was considered deleterious and was located within the fourth transmembrane region of the receptor. Four additional SNPs were non-deleterious (XP_144122.4, Tas1r3, BC002216, and Ttll10), while functionality could not be determined for seven further SNPs (Ccnl2, Mxra8, Tas1r3, and Ube2j2). The deleterious Ile706Thr mutation in Tas1r3 was found in all low sucrose preferring strains for which sequence is available in our database (129X1/J, 129S1/SvImJ, DBA2/J, and A/J). Interestingly, the Ile706Thr mutation in Tas1r3 was not previously reported by researchers studying the sucrose preference QTL, while the Thr55Ala and Ile60Thr mutations previously proposed to contribute to the QTL [32] were either not present in the public databases or predicted to be non-deleterious, respectively. Thus, our analysis suggests that the previously undescribed Ile706Thr transmembrane mutation may contribute to, or even be the primary variation underlying the sucrose preference QTL. These findings demonstrate that our database is able to correctly identify a previously identified candidate gene within a 1.2 Mb QTL interval containing over 50 genes. Furthermore, identification of a putative functional SNP underlying the QTL was achieved without the need for laborious congenic mapping or locus sequencing.

Next, we used Mouse SNP Miner to derive candidate genes for a previously uncharacterized QTL affecting morphine consumption [34]. F2 and recombinant inbred mapping experiments between C57BL/6 and DBA/2 strains were used to identify a 29 Mb QTL that lies between D10Mit3 and the distal tip of chromosome 10 and influences preference for morphine over quinine. These data were confirmed by data showing that congenic B6.D2 mice in which a 28 Mb fragment from the distant tip of chromosome 10 from DBA/2 was introgressed onto C57BL/6J showed morphine consumption resembling DBA/2 [34]. Using Mouse SNP Miner, we retrieved 22 putative functional SNPs differing between C57BL/6J and DBA/2J and lying between 0 and 28,841,602 bp on chromosome 10. Of these 22 SNPs located in 14 transcripts, 14 were considered non-deleterious, 3 were considered deleterious, and 5 were unknown. One of the deleterious mutations (Arg274Gln) was located in an intronless mouse heat shock-related protein, Q3UBR0, in the DBA/2J strain. Variations in this gene are unlikely to contribute to the morphine QTL because its mRNA was absent from mouse tissues according to Symatlas. The remaining deleterious mutations were found in the orphan G-protein coupled receptor, Gpr126 (Gly196Asp), and synaptic nuclear envelope protein 1, Syne1 (Arg386Gln), with the deleterious variant in both cases found in DBA/2J. The mutation in Syne1 lies within a splice variant expressed in cardiac and skeletal muscle, but not brain, and is thus not likely to contribute to the QTL [35]. Gpr126 is a member of the adhesion family of GPCRs [36] and is specifically expressed in placenta, fetal lung and liver, and olfactory epithelium where it could contribute to alterations in olfactory perception. These findings lead us to propose that Gpr126 is a candidate gene for the morphine consumption QTL and demonstrate the power of our database to rapidly screen through SNPs within large QTL to derive candidate genes for further testing. However it is important to point out that the retrieval of candidate SNPs using Mouse SNP Miner is necessarily limited by the extent of sequence coverage for the strains selected and in the particular chromosome regions studied. Although sequencing coverage is rapidly improving, in some cases coverage is still very poor and in these cases candidate genetic variations are likely to be overlooked. It is also important to reiterate the fact that genetic variation other than overtly detrimental coding sequence SNPs contribute to QTL phenotypes. At the moment such variations are not included in the Mouse SNP Miner database. Moreover, care must be exercised when interpreting mRNA expression levels from Symatlas, as microarray data is particularly prone to false negative results.

## Discussion

A large public effort to determine the complete sequences of over 15 inbred mouse strains is presently underway [37]. The inclusion of these data into future versions of our database will dramatically increase its utility and versatility. In addition the further incorporation of new genomic sequences from related species will help improve the power of the PolyPhen and PANTHER algorithms to estimate functionality of amino acid substitutions, as this process for the most part relies on sequence homology. Several additional features of our database could warrant improvement. First, links between SNPs and Symatlas expression data for the relevant transcript in our current database version rely on Ensembl stable transcript IDs. Due to frequent changes in Ensembl IDs, in some cases we failed to retrieve expression data even when the data existed in Symatlas. A referencing system using Affymetrix probes could circumvent this problem and in addition would provide probe-specific expression data that could be correlated with SNP position in the transcript. Second, it is likely that functional non-coding mutations also contribute significantly to QTL phenotypes [2]. The functional consequence of genetic variation in gene regulatory elements, for example, could be assessed using information from transcription factor binding site databases, and such information could be incorporated into future versions of Mouse SNP Miner. Third, the inclusion of data from additional missense SNP functional prediction algorithms, such as SIFT [38], could be envisioned. Like PolyPhen and PANTHER, SIFT uses sequence conservation to predict functional consequences, but differs in the way it assembles protein alignments. Fourth, as additional re-sequencing data becomes available and incorporated into Mouse SNP Miner, false positive SNPs due to sequencing errors and false negative SNPs due to incomplete genome sequence will diminish. Moreover, the inclusion of sequence from additional inbred strains will assure that a larger fraction of known QTL will become amenable to study using our database. Finally, we are aware that in some cases amino acid calls derived from our translation of Ensembl transcripts contain errors due to transcript direction. Ensembl v41 (October 2006) includes amino acid strain assignments for missense SNPs and should allow us to correct this deficit.

## Conclusion

The Mouse SNP Miner database contains mouse SNPs predicted to cause missense, STOP-gain, STOP-lost, frameshift, and splice-site mutations. The database provides several annotations for each SNP, including PolyPhen and PANTHER predictions of missense mutation consequence and gene expression data from Symatlas. Our database allows convenient searching of mouse functional SNPs by strain, chromosomal location, type, predicted functional consequence, gene expression, GO and OMIM terms. The database provides an overview of the extent of functional coding sequence variation between mouse inbred strains and will help to speed the identification of candidate genetic variations that underlie mouse QTL.

## Availability and requirements

The database is freely available at  and requires Java version 1.4 or greater. The web site has been optimized using a PC running the Firefox 1.5 browser, although other platforms are supported as well.

## Authors' contributions

CG and ER conceived of the database; CG participated in its design and coordinated the work; ER designed and assembled the database and web interface and processed the PANTHER algorithm; VR carried out the PolyPhen analysis of mouse sequences; CG and ER drafted the manuscript. All authors read and approved the final manuscript

## Figures and Tables

**Table 1:** Classification of single nucleotide polymorphisms (SNPs) derived from the comparison of four mouse inbred strains.

**Strains**	**SNP Consequence**	**Total SNPs (%)**	**Genes Effected(%)**
**C57BL/6J vs. DBA/2J**	non-synonymous	9,079 (96.6%)	2,956 (90.3%)
	stop-gained	153 (1.7%)	153 (4.7%)
	stop-lost	26 (0.3%)	26 (0.8%)
	splice-site	129 (1.4%)	129 (4%)
	frameshift	13 (0.2%)	13 (0.4%)
	Total	9,400	3,277
			
**C57BL/6J vs. 129S1/SvImJ**	non-synonymous	4,084 (96.5%)	1,597(92.1%)
	stop-gained	73 (1.8%)	67 (3.9%)
	stop-lost	7 (0.2%)	7 (0.5%)
	splice-site	68 (1.7%)	63 (3.7%)
	frameshift	1 (0.1%)	1 (0.1%)
	Total	4233	1,735
			
**C57BL/6J vs. A/J**	non-synonymous	12,435 (96.2%)	3,653 (90.7%)
	stop-gained	262 (2.1%)	191 (4.8%)
	stop-lost	22 (0.2%)	21 (0.6%)
	splice-site	182 (1.5%)	154 (3.9%)
	frameshift	27 (0.3%)	11 (0.3%)
	Total	12,928	4,030

Total number and frequency of Ensembl predicted missense, stop-gained, stop-lost, frameshift, and splice site SNPs for C57BL/6J vs. DBA/2J, C57BL/6J vs. 129S1/SvImJ, and C57BL/6J vs. A/J.

**Table 2:** Summary of PolyPhen and PANTHER annotations of missense SNPs

**Strains**		**Functional Predictions**	**Total SNP(%)**	**Genes Affected(%)**
**C57BL/6J vs. DBA/2J**	**PolyPhen**	Deleterious	1,755 (19.4%)	954 (26.1%)
		Benign	6,757 (74.5%)	2,343 (64%)
		Unknown	567 (6.3%)	367 (10.1%)
		Total	9,079	3,664
				
	**PANTHER**	Deleterious	1,285 (14.2%)	632(17.1%)
		non-deleterious	4,678 (51.6%)	1,736 (47%)
		Unknown	3,116 (34.4%)	1,329 (36%)
		Total	9,079	3,697
				
**C57BL/6J vs. 129S1/SvImJ**	**PolyPhen**	Deleterious	796 (19.5%)	480 (24.9%)
		Benign	2,995 (73.4%)	1,270(65.7%)
		Unknown	293 (7.2%)	185 (9.6%)
		Total	4,084	1,935
				
	**PANTHER**	Deleterious	568 (14%)	327(16.9%)
		non-deleterious	2,049 (50.2%)	913 (47.1%)
		Unknown	1,467 (36%)	701 (36.2%)
		Total	4,084	1,941
				
**C57BL/6J vs. A/J**	**PolyPhen**	Deleterious	2,350 (18.9%)	1,197(26.1%)
		Benign	9,220 (74.2%)	2,889 (63%)
		Unknown	865 (7%)	504 (11%)
		Total	12,435	4,590
				
	**PANTHER**	Deleterious	1,785 (14.4%)	798(17.1%)
		non-deleterious	6,443 (51.9%)	2,154(46.1%)
		Unknown	4,207 (33.9%)	1,724 (36.9%)
		Total	12,435	4,676

According to PolyPhen, 20% of missense mutations contained in the database are predicted to be deleterious (either 'possibly' or 'probably' damaging) to protein function. According to PANTHER, 14% of missense mutations contained in the database are predicted to be deleterious to protein function.

**Table 3:** 

**PolyPhen**	**Deleterious**	**Not-deleterious**	**Not predicted**
PANTHER			
Deleterious	1,647	2,092	73
not-deleterious	1,857	11,569	310
Not predicted	1,818	5,888	1,889

PolyPhen and PANTHER predictions overlap significantly, with 6.1% of missense mutations categorized as detrimental by both algorithms.

**Table 4:** Summary of OMIM annotations of predicted functional SNPs.

**Overall SNP Consequence**	**Redundant Genes Effected**	**OMIM Entries**
non-synonymous (damaging SNPs)	803	859
stop-gained	90	99
stop-lost	14	15
splice-site	114	116
frameshift	36	37

Greater than 15% of genes containing at least one predicted functional SNP (splice site, frame shift, STOP-gain, STOP-lost, deleterious missense according to Polyphen) in the database have human orthologs found in the OMIM database of disease-associated mutations.
